# Muc5ac gastric mucin glycosylation is shaped by FUT2 activity and functionally impacts *Helicobacter pylori* binding

**DOI:** 10.1038/srep25575

**Published:** 2016-05-10

**Authors:** Ana Magalhães, Yannick Rossez, Catherine Robbe-Masselot, Emmanuel Maes, Joana Gomes, Anna Shevtsova, Jeanna Bugaytsova, Thomas Borén, Celso A. Reis

**Affiliations:** 1 i3S - Instituto de Investigação e Inovação em Saúde, Universidade do Porto, Rua Júlio Amaral de Carvalho, 45, 4200-135 Porto, Portugal; 2 Institute of Molecular Pathology and Immunology of University of Porto, Ipatimup, Rua Júlio Amaral de Carvalho, 45, 4200-135 Porto, Portugal; 3 Structural and Functional Glycobiology Unit, UMR CNRS 8576, University of Lille, 59655 Villeneuve d’Ascq, France; 4 Department of Medical Biochemistry and Biophysics, Umeå University, SE-901 87 Umeå, Sweden; 5 Medical Faculty, University of Porto, Al. Prof. Hernâni Monteiro, 4200–319 Porto, Portugal; 6 Instituto de Ciências Biomédicas de Abel Salazar (ICBAS), University of Porto, Rua de Jorge Viterbo Ferreira no 228, 4050-313 Porto, Portugal

## Abstract

The gastrointestinal tract is lined by a thick and complex layer of mucus that protects the mucosal epithelium from biochemical and mechanical aggressions. This mucus barrier confers protection against pathogens but also serves as a binding site that supports a sheltered niche of microbial adherence. The carcinogenic bacteria *Helicobacter pylori* colonize the stomach through binding to host glycans present in the glycocalyx of epithelial cells and extracellular mucus. The secreted MUC5AC mucin is the main component of the gastric mucus layer, and BabA-mediated binding of *H. pylori* to MUC5AC confers increased risk for overt disease. In this study we unraveled the *O-*glycosylation profile of Muc5ac from glycoengineered mice models lacking the FUT2 enzyme and therefore mimicking a non-secretor human phenotype. Our results demonstrated that the FUT2 determines the *O-*glycosylation pattern of Muc5ac, with Fut2 knock-out leading to a marked decrease in α1,2-fucosylated structures and increased expression of the terminal type 1 glycan structure Lewis-a. Importantly, for the first time, we structurally validated the expression of Lewis-a in murine gastric mucosa. Finally, we demonstrated that loss of mucin FUT2-mediated fucosylation impairs gastric mucosal binding of *H. pylori* BabA adhesin, which is a recognized feature of pathogenicity.

The mucosal epithelial cells of the gastrointestinal tract are covered by an extracellular mucus layer, which constitutes an important physical barrier against external aggressions such as chemical agents, microorganisms and mechanical stress^1^. The major components of this gel are water and heavily glycosylated secreted mucins. Importantly, the large diversity of glycan epitopes carried by mucins^2^ provides binding sites for commensal microorganisms^1^ but also for pathogens, including the gastric carcinogenic bacteria *Helicobacter pylori* (*H. pylori*)^3^,^4^.

*H. pylori* is one of the most common human infectious agents and *H. pylori* infection is considered the main cause of gastric cancer^5^,^6^. *H. pylori* is mostly found within the mucinous layer that covers and protects the gastric epithelium^7^,^8^. The major constituents of the gastric mucus layer are the secreted MUC5AC mucin, produced by the surface mucus-secretor cells and MUC6, secreted by the deeper glands^9^,^10^. Structural analysis of the *O*-glycans carried by gastric mucins has revealed a major expression of core 2-based sequences [Galβ1–3(GlcNAcβ1–6)GalNAc-]^11^,^12^. Furthermore, detailed structural characterization of the terminal glycan epitopes carried by MUC5AC mucin showed dominating H blood group structures [Fucα1,2Galβ1,3(4)GlcNAc-] and structures containing the di-N-acetyllactosamine (lacdiNAc) unit (GalNAcβ1,4GlcNAcβ1-)^11^. Similarly, structural characterization of the glycans carried by the murine Muc5ac mucin has demonstrated that the most abundant structures were based on core 2 sequences^13^. However, in contrast to human MUC5AC, termination with histo blood group antigens was not so frequently found in mice^14^.

*H. pylori* colonization of the stomach is achieved by interaction of bacterial outer-membrane proteins, that present lectin binding properties, with host glycan receptors expressed in the gastric mucus layer and in the glycocalyx of mucosal epithelial cells^3^. The Blood group antigen binding adhesin (BabA) mediates binding to the ABO(H)/Lewis b blood group antigens^15^,^16^. BabA-mediated binding to MUC5AC is recognized as an important mode of *H. pylori* adhesion and BabA expression is a virulence factor associated with increased risk for overt disease^17^,^18^,^19^. *H. pylori* binding to MUC5AC may also be achieved through recognition of lacdiNAc motifs by the LacdiNAc-specific adhesin (LabA)^20^. Upon *H. pylori* infection, the gastric mucosa inflammation is accompanied by a shift in the gastric glycophenotype from expression of neutral structures to *de novo* expression of α2,3-sialylated glycans^21^,^22^,^23^,^24^, which constitute ligands for the Sialic acid binding adhesin (SabA)^23^. This shift into a more sialylated mucosal landscape during *H. pylori* infection is driven by up-regulation of specific glycosyltransferases, whose increased expression levels result in increased biosynthesis of sialylated Lewis glycan structures^21^,^22^.

The mucins glycosylation pattern is plastic and dynamic and defined by the enzymatic activity of several glycosyltransferases, and the gastric mucins glycosylation profile reveals important roles for the core 2 β1,6-N-acetylglucosaminyltransferase (C2GnT) and the α1,2-fucosyltransferase (FUT2) enzymes in the biosynthesis of its *O-*glycan structures. Therefore, genetic variations in the genes encoding specific glycosyltransferases result in diversity of mucin glycan structures. The FUT2 enzyme catalyzes the addition of terminal α1,2-fucose residues driving the biosynthesis of H type 1 structures, which *H. pylori* can attach to, on gastrointestinal cells and mucosal secretions of secretor individuals (Fig. 1a)^25^. Inactivating mutations on the *FUT2* gene are associated with a non-secretor phenotype and result in inability to produce α1,2-fucosylated type 1 sequences in neither body secretions nor in the gastrointestinal lining. Thus, secretor individuals express ABO/Lewis b blood group antigens in secretion and in the gastrointestinal tract epithelium, in contrast to non-secretors. The non-secretor phenotype is found almost exclusively in Europe and constitutes 20% of the Caucasian population. This is of particular clinical relevance, since the individuals secretor status affects *H. pylori* infection susceptibility^26^,^27^. In agreement, we have shown that Fut2-null mice, an animal model of non-secretors, present impaired BabA-mediated *H. pylori* adhesion^28^.

Tissue profiling by immunohistochemistry has shown expression of Lewis a (Galβ1–3(Fucα1,4)GlcNAcβ1) and Lewis b (Fucα1,2-Galβ1–3(Fucα1,4)GlcNAcβ1) in mice gastric mucosa^28^. However, structural analysis of total *O-*glycans from mice gastric samples had never yielded evidence for these type 1 structures, most probably due to their low abundance in comparison with type 2 sequences^14^,^28^,^29^. Knowing that the MUC5AC is the major carrier of type 1 Lewis structures in human gastric mucosa^17^,^18^,^30^, in this study we have evaluated whether the mouse orthologue Muc5ac presented similar glycosylation features. We have used, as comparative experimental models, wild-type mice and the Fut2 null mice that mimic the human secretor and non-secretor gastric glycosylation profiles. The expression pattern of Muc5ac and Lewis glycan antigens in mice gastric tissue was characterized and comprehensive structural analysis of the Muc5ac glycosylation profile was performed with a focus on *O*-glycans carrying Lewis determinants. Finally, we evaluated the functional impact of loss of mucin FUT2-mediated fucosylation in *H. pylori* BabA binding to the murine gastric mucosa.

## Results

### Muc5ac is modified with Lewis b-terminated glycan structures in wild-type “secretor” mice gastric tissue

**Figure 1:**
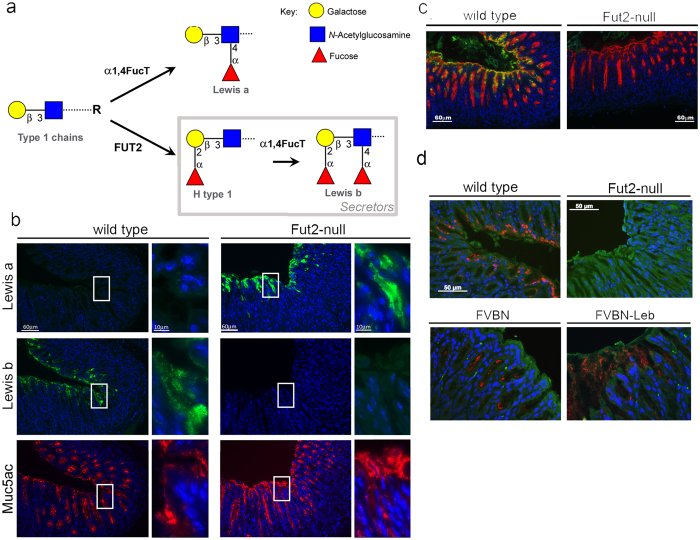
Muc5ac and Lewis b-terminated glycan structures are expressed in close molecular proximity in “secretor” wild-type mice. (**a**) Schematic representation of the biosynthetic pathway leading to type 1 Le^a^ and Le^b^ antigens expression. Paraffin-embedded sections of wild-type and Fut2-null mice gastric mucosa, fixed with Carnoy’s solution, were used for: (**b**) immunofluorescence labeling with Le^a^ (7LE), Le^b^ (BG6) and Muc5ac (45M1) recognizing antibodies, images on the right present higher magnifications of the areas within the white rectangles; (**c**) double-immunofluorescence labeling with Muc5ac and Le^b^ recognizing antibodies, using fluorescent-labeled immunoglobulin subtype specific secondary antibodies; and (**d**) *in situ* Proximity Ligation Assay (PLA) for Muc5ac and Le^b^. Each fluorescent red spot corresponds to a PLA signal and is indicative that Muc5ac and Le^b^ are in close proximity. FVBN wild-type and FVBN-LeB transgenic mice gastric mucosa tissue sections were also included in PLA analysis as experiment biological positive controls. All tissue sections were stained with DAPI for nucleus visualization.

No major differences were observed regarding the pattern of Muc5ac expression in wild-type and Fut2-null mice, Muc5ac expression was restricted to the surface foveolar epithelium of both mice secretor and non-secretor phenotypes (Fig. 1b). In agreement with our previous observations^28^, we observed Le^b^ expression in the superficial mucous cells of wild-type mice but not the gastric mucosa of “non-secretor” Fut2-null mice (Fig. 1b). Reciprocally, Le^a^ was expressed in the surface cells of Fut2-null mice but not in wild-type mice gastric mucosa (Fig. 1b). Double-labelling analysis showed that Le^b^ antigen was co-expressed with the Muc5ac mucin at the surface foveolar epithelium of wild-type mice (Fig. 1c). To test if Muc5ac is the carrier of the Le^b^ glycan in mice gastric mucosa, we applied *in situ* Proximity Ligation Assay (PLA) using the Le^b^ (BG6) and Muc5ac (45M1) recognizing antibodies. In wild-type mice gastric mucosa we observed a complete overlap of the PLA signals with the co-expression of Muc5ac and Le^b^ detected by double-immunofluorescence, showing that wild-type Muc5ac is modified with Le^b^-terminated glycan structures recognized by the BG6 antibody (Fig. 1c,d). As expected, no PLA signals were observed in Fut2-null mice gastric tissue. As proof of concept for the PLA experiment, we evaluated gastric mucosa from the Le^b^ transgenic mouse (FVB/N Leb), that overexpress Le^b^ in the gastrointestinal tract, and the corresponding wild-type mice (FVB/N). The extent and intensity of PLA signals in the gastric mucosa of the Le^b^-overexpressing mice model (FVBN-Leb) was, as expected, much higher compared to the control FVBN wild-type (Fig. 1d).

### Lack of FUT2 enzyme results in an enrichment of Lewis a glycan structures

**Figure 2:**
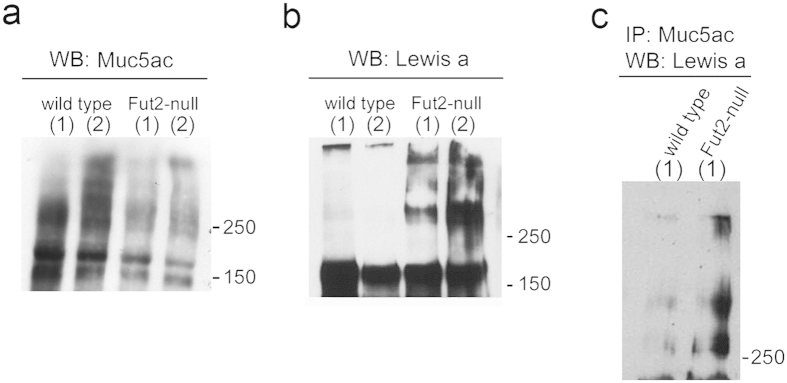
Muc5ac from “non-secretor” Fut2-null mice displays enrichment in Lewis a glycan structures. Wild-type and Fut2-null mice total gastric mucosa protein lysates were used for evaluation of (**a**) Muc5ac (45M1) and (**b**) Le^a^ (SPM279) expression by Western blotting. Labels (1) and (2) represent protein lysates from two independent mice samples. (**c**) G-sepharose beads coupled with Muc5ac recognizing antibody 45M1 were incubated with wild-type and Fut2-null mice gastric mucosa protein extracts. Proteins that bound to the beads were analyzed by Western blotting with an anti -Le^a^ antibody.

Le^a^ expression in mice gastric mucosa was evaluated using total protein extracts from gastric mucosal samples. Immunoblot analysis of Muc5ac (45M1) expression showed for both wild-type and Fut2-null mice a high-molecular-weight (>200 kDa) smear, which is characteristic of highly glycosylated mucins (Fig. 2a). Although the same amount of protein was loaded for wild-type and Fut2-null mice, Muc5ac immunoreactivity was higher in wild-type mice. This difference may stem from differences in the Muc5ac glycosylation profile. Evaluation of the *Muc5ac* and *Muc6* transcript levels showed no differences in the expression of these gastric mucins when comparing wild-type and Fut2-null mice (Supplementary Fig. S1). In accordance with the results of Le^a^ histoprofiling in gastric mucosa, we observed higher expression of Le^a^ in the Fut2-null mice gastric mucosal lysates (Fig. 2b). However, some Le^a^ immunoreactivity was observed in the wells loaded wild-type mice extracts, suggesting the presence of high-molecular weight mucins modified with Le^a^ also in wild-type mice. Immunoblots of immunoprecipitated Muc5ac confirmed that Le^a^ was higher expressed in the Fut2-null mice (Fig. 2c).

### Muc5ac from “secretor” and “non-secretor” mice display distinct expression patterns of Lewis type 1 glycan structures

**Figure 3:**
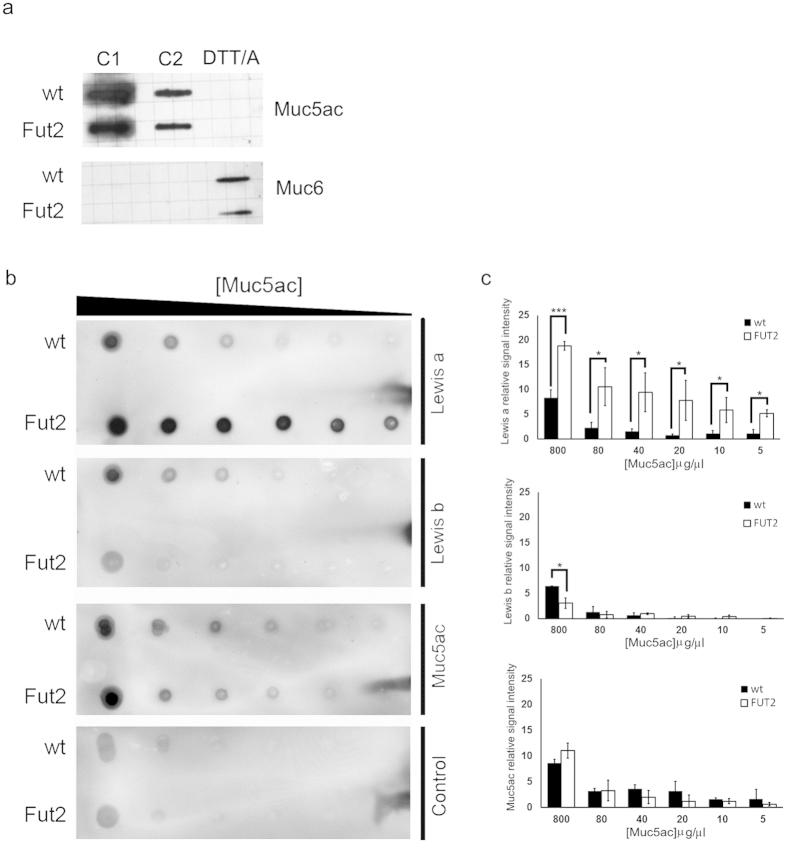
Lack of FUT2 results in altered Muc5ac glycosylation patterns. (**a**) Slot-blot analysis of collected supernatant fractions (C1, C2) and pellet fraction, upon solubilization by DTT/alkylation (DTT/A) treatment, using the LUM5-1 and LUM6-3 antibodies for Muc5ac and Muc6, respectively. (**b**) Serial dilutions (800; 80; 40; 20; 10 and 5 μg/μl) of purified Muc5ac from wild-type and Fut2-null mice gastric samples were probed with Le^a^ (SPM279) and Le^b^ (BG6) recognizing antibodies. Muc5ac loading amounts were verified using the PM7 mixture of antibodies (Table 1). A negative control, without primary antibody incubation, was included for detection of background staining of non-specific secondary antibody binding. (**c**) Quantification of dot blot relative intensity. The optical density of each signal was measured in three independent dot-blot analyses. Densitometry average values with standard deviation are shown.

In order to structurally characterize *O-*glycans carrying Lewis determinants the gastric Muc5ac was purified by density-gradient centrifugation. The slot blot analysis shown in Fig. 3a demonstrates an efficient separation of isolated Muc5ac and Muc6 fractions. A screening dot blot analysis of purified Muc5ac from wild-type and Fut2-null mice revealed different patterns of immunoreactivity using type 1 Lewis recognizing antibodies. Le^a^ (SPM279) immunoreactivity was significantly higher in the full serial dilutions of Muc5ac from Fut2-null mice, when compared with wild-type mice samples (Fig. 3b,c). In contrast, the Le^b^ recognizing antibody (BG6) showed higher immunoreactivity with Muc5ac purified from wild-type mice than Muc5ac from Fut2-null mice (Fig. 3b,c).

### Gastric Muc5ac is a carrier of Lewis a in mice lacking FUT2

**Figure 4:**
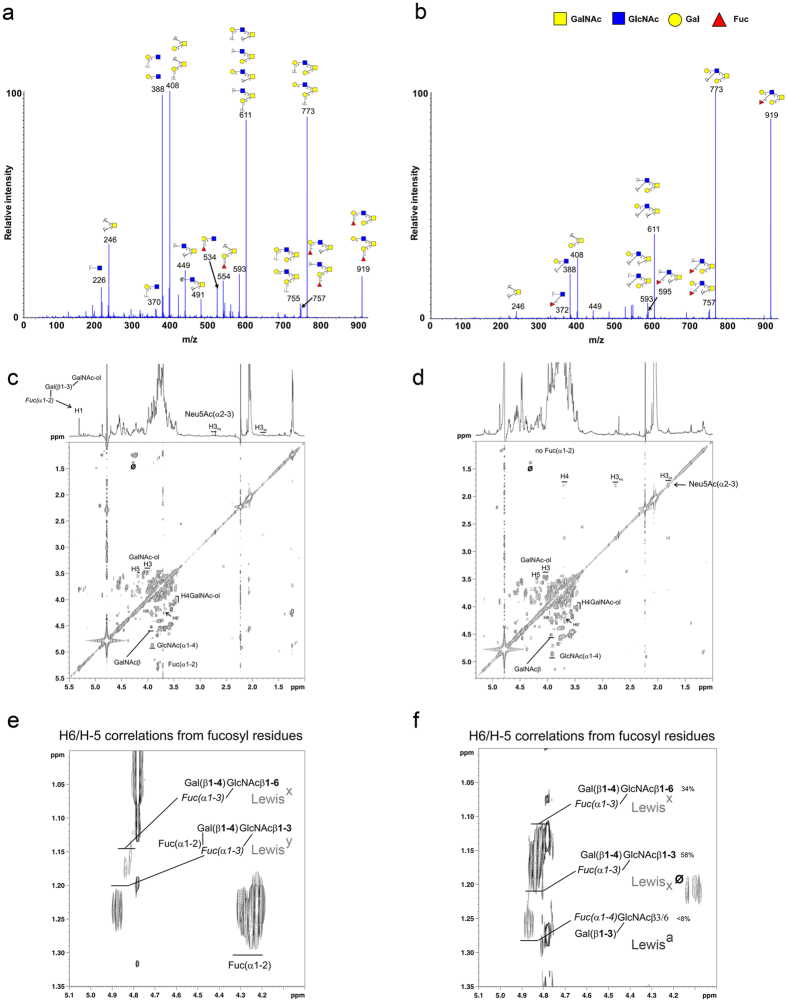
Structural analysis of the glycan structures carried by Muc5ac in wild-type and Fut2-null mice gastric mucosa. MS/MS spectra of the pseudomolecular ion at m/z 919, isolated from gastric MUC5AC mucins from (**a**) wild-type and (**b**) Fut2-null mice, acquired in the positive ion mode. Nuclear magnetic resonance analysis of (**c**,**e**) wild-type and (**d**,**f**) Fut2-null mice for determination of the glycan structures present on the gastric Muc5ac mucin. Part of ^1^H-^1^H 2D-NMR COSY spectrum acquired on a 14.3Teslas spectrometer of total *O-*glycans isolated from gastric mucin of (**c**) wild-type and (**d**) Fut2-null mice Muc5ac mucin. (**e**) Extended part of the wild-type even COSY spectrum showing the chemical shifts zone of H6/H5 correlation from fucose residues. (**f**) Extended part of the Fut2-null mice NMR COSY spectrum shown in panel (**d**).

Oligosaccharides were released by base/borohydride treatment and analyzed by nano-ESI quadrupole time-of-flight (Q-TOF) and NMR spectroscopy. As previously described^28^,^29^, almost all the gastric *O*-glycans from wild-type mice were based on a core 2 structures and carried blood group H determinants (Fucα1–2Galβ1–3/4-R), whereas most of the *O*-glycans from Fut2-null mice lack blood group H antigen. Fucose residues were mainly carried by N-acetylglucosamine residues, constituting Le^a^ or Le^x^ determinants. For example, the molecular ion at m/z 919 in the positive ion mode, which is present in both wild-type and Fut2-null samples, corresponded to different fucosylated structures (Fig. 4a,b). In wild-type samples, MS/MS spectrum of this ion showed two B_i_ ions at m/z 331 and 534, corresponding respectively to a disaccharide Fucα1–2Gal and the trisaccharide Fucα1–2Galβ1–3/4GlcNAc, consistent with the presence of the H-antigen. In contrast, the MS/MS spectrum of the ion at m/z 919 recovered in Fut2-null samples was characterized by the presence of fragment ions at m/z 372 and 595, demonstrating that fucose was linked to a *N*-acetylglucosamine residue, consistent with the expression of a Le^a^ or Le^x^ determinant. In order to structurally confirm that gastric MUC5AC from Fut2-null mice carried Le^a^ epitopes, NMR spectroscopy experiments were conducted on the mixture of oligosaccharide-alditols obtained from the two types of samples (Fig. 4c–f). The main anomeric protons observed on the ^1^H-^1^H 2D-NMR COSY spectrum of total *O-*glycans isolated from gastric mucin of wild-type mice showed the presence of two types of *N*-acetyl hexosamine, *i.e.* GlcNAc and GalNAc, and one fucose residue. This fucose entity is *O-*2 linked since it possessed a typical anomeric chemical shift at around 5.3 ppm. Moreover, correlations related to sialic acid were observed at 1.8 and 2.7 ppm corresponding to H3ax and H3eq respectively; indicating the presence of an α2–3 linked sialic acid (Fig. 4c). Extended part of the even COSY showing the chemical shifts zone of H6/H5 correlation from fucose residues reveals NMR traces characteristic and typical of both Le^x^ and Le^y^ as well as α1,2 fucosylated H structures (Fig. 4e). The ^1^H-^1^H 2D-NMR COSY spectrum of total *O*-glycans isolated from Fut2-null mice gastric mucin shows unambiguously total absence of fucose *O*-2 linked residue since no anomeric signal is observed. Sialic acid was identified throughout the partial spin system H3ax, H3eq and H4 (Fig. 4d). By comparison with wild-type, it is possible to observe in the extended part of Fut2-null NMR COSY spectrum, shown in Fig. 4f, the presence of Le^x^ with a fucose substituting a GlcNAcβ1–3 or a GlcNAcβ1–6 linked. In addition, it was also possible to identify type 1 Le^a^ structures as proved by the H6/H5 NMR signals related to fucose units. No trace of *O*-2 linked fucose was observed. A quantitative estimation of both Lewis structures indicated that Le^x^ represented around 92% of total Lewis antigens whereas Le^a^ represented less than 8% (Fig. 4f) in Muc5ac from Fut2-null mice.

### *Helicobacter pylori* shows impaired BabA-mediated binding to Fut2-null Muc5ac

**Figure 5:**
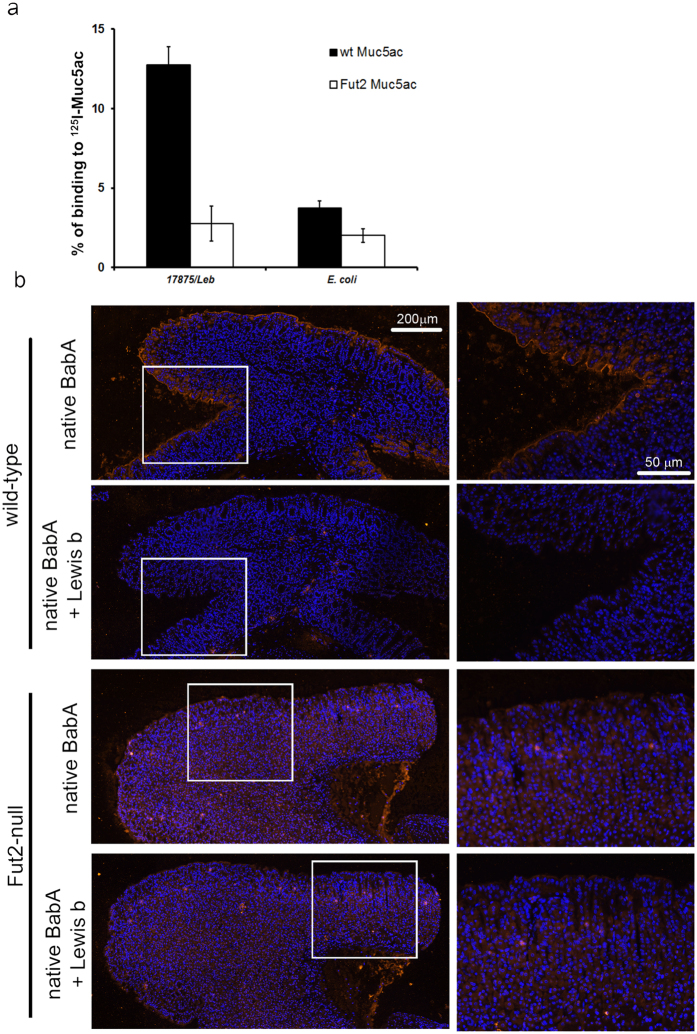
Functional impact of altered Muc5ac glycosylation on *Helicobacter pylori* binding. (**a**) Binding of *H. pylori* reference strain 17875/Leb (BabA+/SabA−) to purified Muc5ac from wild-type and Fut2-null mice labeled with ^125^I was tested by radio immuno assay (RIA). The y axis gives the percentage of bacteria that bound to radio labeled Muc5ac. *E. coli* was included in the assay to test for non-specific bacterial binding to Muc5ac. (**b**) *H. pylori* blood group antigen binding adhesin (BabA) histobinding to wild-type and Fut2-null mice gastric mucosa. Paraffin-embedded sections of wild-type and Fut2-null mice gastric mucosa, fixed with Carnoy’s solution, were incubated with native BabA adhesin isolated from 17875/Leb strain. BabA binding to the mice tissue sections was detected by immunofluorescent labeling with an anti-BabA specific secondary antibody. Sequential mice tissue sections were probed with BabA that was pre-incubated with its cognate ligand Le^b^, to assess the specificity of BabA mucosal binding. Images on the right represent higher magnifications of the regions within the white rectangles.

Knowing that *H. pylori* binds to human MUC5AC in a Le^b^-dependent manner^17^,^18^, we evaluated whether the different glycosylation pattern of Muc5ac from “non-secretor” Fut2-null mice would impact *H. pylori* binding. Binding was tested using the reference *H. pylori* strain 17875/Leb which has been previously characterized regarding BabA expression and binding activity^15^,^23^,^31^. The 17875/Leb strain expresses a functional BabA adhesin and binds the ABO/Leb antigens but does not bind sialylated antigens. Bacterial binding activity for Muc5ac was assessed by radio immunoanalysis (RIA) with ^125^I-labeled Muc5ac. As shown in Fig. 5a, *H. pylori* bind efficiently to Muc5ac from wild-type mice (12,8%), in contrast to Muc5ac from Fut2-null mice (2,8%), which is similar to the negative binding control *E. coli* (2,2%).

### BabA adhesin is unable to bind to Fut2-null mice gastric tissue

We have previously shown that BabA-associated *H. pylori* bacterial adherence to gastric mucosa is lost in the Fut2-null mouse^28^. In order to understand if this impairment of adhesion could be strictly attributed to the BabA protein, we evaluated binding of native BabA purified from the 17875/Leb strain to the gastric mucosa of wild-type and Fut2-null mice. BabA binding signal was detected by immunofluorescence using a BabA-specific antibody. As shown in Fig. 5b, native BabA protein bound to the apical membrane of the surface mucous cells of wild-type mice, but did not bind to the gastric mucosa of Fut2-null mice. Pre-incubation of BabA with its cognate receptor Le^b^ prior to contact with the gastric sections completely abolished BabA binding to mice gastric mucosa (Fig. 5b).

## Discussion

Mucins are high molecular weight glycoproteins with critical roles in the interface of mucosal surfaces and exterior environment throughout the body. The secreted mucin MUC5AC is a major component of the mucinous layer lining the gastric epithelium, conferring protection against luminal acid and proteolytic enzymes. The glycosylation of mucins is determined by the concerted enzymatic activity of several glycosyltransferases and modifications on the mucins glycosylation can impact infection susceptibility and disease progression^1^,^2^,^32^. In the present study, we used complementary structural and functional assays and demonstrated that the FUT2 enzyme determines the glycosylation of the murine gastric mucin Muc5ac, with critical implications on bacterial binding to gastric mucosal tissue.

Previous immunohistological and *in situ* hybridization studies have shown that the expression of MUC5AC mucin is restricted to the surface epithelium of the stomach, both in humans and mice^30^,^33^,^34^,^35^. In agreement, using the 45M1 monoclonal antibody, that recognizes the C-terminal cysteine-rich region of the MUC5AC mucin^36^, we observed that Muc5ac expression was exclusive of the surface foveolar epithelium of mice gastric mucosa. In line with previous observations from our group, we observed a significantly different pattern of expression of the type 1 Lewis antigens, Le^a^ and Le^b^, in “secretor” wild-type and “non-secretor” Fut2-null mice^28^ (Fig. 1a). Whereas the Le^b^ expression was exclusively observed in wild-type mice, Le^a^ expression was only observed at the surface epithelium of Fut2-null mice. Noteworthy, and similar to what has been described for human gastric mucosa^30^,^37^, the expression of Le^a^ and Le^b^ antigens in mice gastric mucosa was restricted to the surface mucous cells (Fig. 1), strongly suggesting Muc5ac as a key carrier of these structures. In order to confirm this hypothesis, a series of complementary biochemical assays were performed. Double immuno-labeling showed co-expression of Muc5ac and Le^b^ in wild-type mice, which, as expected, was lost in Fut2-null mice. To validate this data *in situ* PLA was performed and clearly demonstrated that Muc5ac and Le^b^ co-localize in mice gastric mucosa (Fig. 1). Moreover, immunoprecipitation of Muc5ac from Fut2-null mice total gastric mucosa protein extracts was accompanied by Le^a^ detection (Fig. 2). Altogether, these results support the hypothesis of Muc5ac being a carrier of type 1 Lewis antigens in mice gastric mucosa.

Since structural analysis of total *O*-glycans from murine whole stomach or mucosal scrapings samples had always failed to identify type 1 Lewis antigens^14^,^28^,^29^, we took advantage of the Fut2-null mice model that presents a simplified gastric glycosylation profile, due to massive loss of fucosylated glycans, and performed structural analysis of *O*-glycans released from purified Muc5ac. A first screening of the glycosylation profile of purified Muc5ac confirmed the immunohistochemistry and biochemical assays by revealing that Muc5ac from Fut2-null mice showed higher expression of Le^a^ whereas Le^b^ was exclusively expressed by Muc5ac from wild-type mice (Fig. 3).

Structural analysis of the glycan structures carried by Muc5ac by electrospray mass spectrometry (nano ESI-MS/MS) identified core 2-based structures with terminal blood group H epitope as the dominant structures in wild-type mice. As expected, and as previously described, most of the *O*-glycans from Fut2-null mice showed a major loss of the H antigen (Fig. 4a,b)^28^. Mass spectrometry analysis revealed molecular ions (example m/z 919) with a MS/MS spectrum consistent with the expression of Lewis determinants. Remarkably, further structural characterization by NMR spectroscopy allowed the unequivocal identification of the type 1 Lewis antigen Le^a^ in Fut2-null mice (Fig. 4d,f). In line with previous immunohistochemical evaluation of Lewis antigens expression in mice gastric tissue, the relative abundance of Le^a^ structure was significantly lower (8%) than the abundance of type 2 Lewis antigens, Le^x^ (58% β1,3-linked and 34% β1,6-linked). Besides the positive results by immuno detection for Le^b^ expression, we did not detect type 1 structures in wild-type mice gastric mucosa by structural analysis (Fig. 4c,e). This can be attributed to the very high abundance of α1,2-fucosylated structures in wild-type mice that may hamper the detection of less abundant structures. Further studies would be required in order to characterize the very low abundant glycans structures carried by wild-type gastric mucins. Nevertheless, our results constitute a major finding since they demonstrate for the first time expression of type 1 Lewis antigens in mice gastric mucosa, implying the existence of an enzyme with α1,4-fucosyltransferase activity in mice gastric cells. Mice lack an orthologue of the human *FUT3* gene and no enzyme with α1,4 fucosyltransferase activity has been previously identified in murine gastric tissue^38^,^39^,^40^. Further studies addressing the glycosyltransferases involved in the biosynthesis of type 1 Lewis antigens in mice gastric tissue are warranted.

Mucin glycosylation is reported to differ between individuals and to change accordingly to the pathophysiological status of the individual. The modulation of the mucins glycosylation profile has implications on pathogen recognition and binding capacity. *H. pylori* is found both in the mucus layer and adhering to the gastric epithelium, and *H. pylori* capacity to bind to MUC5AC is well established^17^,^18^. Additionally, binding to MUC5AC has been reported to increase *H. pylori* proliferation^41^. Moreover, it has been shown that the gastric mucins glycosylation profile is altered in response to *H. pylori* infection^32^. In this study, we have evaluated how the different Muc5ac glycosylation patterns presented by wild-type and Fut2-null mice influenced *H. pylori* binding capacity. We observed, by radio immunoassay, that *H. pylori* showed different binding to Muc5ac isolated from wild-type and Fut2-null mice. The BabA-competent strain 17875/Leb adhered significantly more to wild-type Muc5ac than to Muc5ac purified from Fut2-null mice mucosa. Furthermore, we demonstrated specific binding of native BabA adhesin to gastric tissue sections of wild-type mice, whereas binding to Fut2-null mice gastric mucosa was abrogated (Fig. 5).

In summary, we demonstrated that the FUT2 enzyme determines the *O*-glycosylation pattern of Muc5ac, leading to a marked decrease in α1,2-fucosylated terminal structures and increased type 1 Le^a^ expression. Moreover, we demonstrated, for the first time at a structural level, the expression of terminal type 1 Lewis structures in mice gastric mucosa and identified Muc5ac as the carrier of these structures. Finally, we demonstrated that loss of mucin FUT2-driven fucosylation abolished BabA-mediated *H. pylori* adhesion. This biochemical and functional characterization of a non-secretor mice model opens new opportunities for translational research on the impact of the individual’s secretor status in gastrointestinal disease and clinical applications.

## Methods

### Animal models

Wild-type C57BL/6 and Fut2-null mice^42^ were obtained from the Consortium for Functional Glycomics. Mice were housed, reproduced and maintained at IPATIMUP’s Animal House at the Medical Faculty of the University of Porto and handled accordingly to the Guidelines for the Care and Use of Laboratory Animals, directive 2010/63/UE. All experimental procedures were approved by ORBEA (Orgão responsável pelo bem-estar dos animais da Faculdade de Medicina da Universidade do Porto/Committee for animal welfare of the Medical Faculty of the University of Porto) and were performed in accordance with the ethics committee guidelines and regulations, following directive 2010/63/UE. Mice genotyping was performed as previously described, using primers specific for the Fut2 wild-type and the null alleles^28^,^42^.

### Tissue samples

Stomachs from 6–8 weeks-old wild-type and Fut2-null were harvested, washed with 0.9% NaCl solution and immediately frozen or fixed in Carnoy’s solution and embedded in paraffin wax. Tissue sections from FVB/N and FVB/N transgenic mice expressing the human α1,3/4 fucosyltransferase FUT3 (Le^b^ transgenic mouse)^43^ were obtained from formalin fixed gastric mucosa embedded in paraffin wax. Serial sections of 3 μm were cut and used for tissue labeling analysis.

### Immunofluorescence labeling

After deparaffination and rehydration of the tissue sections, according to the standard protocol, tissues were subject to heat-induced antigen retrieval by boiling slides in citrate pH 6.0 solution for 20 min. Samples were then washed twice in PBS and incubated for 20 minutes with goat non-immune serum (DAKO, Glostrup, Denmark) for Muc5ac and Le^a^ labeling, or rabbit non-immune serum (DAKO, Glostrup, Denmark) for Le^b^ labeling, diluted 1:5 in PBS containing 10% of BSA. Tissue samples were incubated overnight at 4 °C with the respective primary antibodies: 45M1 (Muc5ac), 7LE (Le^a^) or BG6 (Le^b^) diluted in PBS with 5% BSA, as described in Table 1. After, sections were washed in PBS and incubated with either Rhodamine-goat anti-mouse IgG1 (Jackson Immunoresearch Laboratories, West Grove, PA, USA) diluted 1:50 in PBS with 5% BSA (Muc5ac), FITC-rabbit anti-mouse Igs (DAKO, Glostrup, Denmark) diluted 1:70 in PBS with 5% BSA (Le^a^) or FITC-goat anti-mouse IgM (Southern Biotech, Birmingham, AL, USA) diluted 1:30 in PBS with 5% BSA (Le^b^) for 1 hr at room temperature. Sections were then washed with PBS and incubated with DAPI. Finally, samples were washed in PBS and mounted in Vectashield (Vector Laboratories, Inc, Burlingame, CA, USA). For double immuno labeling of Muc5ac and Le^b^ a similar protocol was used with the following modifications. After the blocking step with normal goat serum, tissues were incubated with the first primary antibody BG6 (Le^b^) over-night at 4 °C as described above. Then, after the washing steps, sections were incubated with FITC-conjugated goat anti-mouse IgM, diluted 1:30 in PBS containing 5% BSA, for 1 hr. Tissue samples were washed with PBS and incubated with the second primary antibody 45M1 (Muc5ac) over-night at 4 °C as previously described. Then, tissues were incubated for 1 hr with Rhodamine-conjugated goat anti-mouse IgG1, diluted 1:50 in PBS with 5% BSA and DAPI, in the dark. Images were acquired using a Zeiss Axio cam MRm and the AxioVision Rel. 4.8 software.

### Proximity ligation assay (PLA)

PLA assays were performed using the DuoLink II kit (Olink AB, Uppsala, Sweden), according to the manufacturer’s instructions with the following modifications. Tissue sections were subject to deparaffination, rehydration and antigen retrieval procedures as described above for immunofluorescence labeling. Tissue samples were then incubated for 30 minutes with goat non-immune serum, diluted 1:5 in PBS containing 10% of BSA. Then, sections were incubated over-night at 4 °C with BG6 (Le^b^) and 45M1 (Muc5ac) primary antibodies diluted in PBS with 5% BSA (Table 1). Samples were washed twice with PBS and incubated with anti-IgM and anti-IgG oligonucleotide-conjugated PLA secondary probes diluted 1:10 in PBS with 5% BSA in a pre-heated humid chamber for 1 hr and 45 minutes at 37 °C. The anti-IgM and anti-IgG probes were prepared using AffiniPure Goat antimouse IgG or IgM (Jackson Immunoresearch Laboratories, West Grove, PA, USA) secondary antibodies, as previously described^44^. Ligation and Amplification steps were performed according to manufactures’ instructions. Rolling circle products were visualized with fluorescently labeled oligonucleotides and slides were mounted using Duolink II Mounting Medium with DAPI. Samples were examined under a Zeiss Imager.Z1 Axio fluorescence microscope. Proximity ligation assays products are seen as bright fluorescent dots. Images were acquired using a Zeiss Axio cam MRm and the AxioVision Rel. 4.8 software. Double-immunostaining and PLA analysis of mice gastric mucosa using the Muc5ac and Le^a^ recognizing antibodies pair were not possible since the primary antibodies available are from the same immunoglobulin subtype.

### Western blotting and Muc5ac Immunoprecipitation

Gastric mucosa total protein extracts were prepared using a non-denaturating lysis buffer (1% w/v Triton X-100, 50 mM Tris-HCl pH 7.4, 300 mM NaCl, 5 mM EDTA, 0.02% w/v sodium azide, 10 mM iodoacetamide, 1 mM PMSF (phenylmethanesulfonyl fluoride), 1 mM Na_3_VO_4_ and protease inhibitor cocktail). Protein concentration was determined using the BCA protein assay kit (Pierce, Thermo Scientific, Rockford, IL, USA). For Le^a^ and Muc5ac Western blotting analysis, 50 μg of total protein lysate were loaded in an acrylamide gel (stacking 4%/resolving 5%), under non-reducing conditions for electrophoresis, and Western blotting was performed as previously described using the 45M1 (1:1000) antibody for Muc5ac and SPM279 (sc-52988 Santa Cruz) (1:200) for Le^a^ detection^28^. For Muc5ac immunoprecipitation, equal amounts of total protein (750 μg) from either wild-type or Fut2-null mice lysates were precleared with 25 μL of protein G-sepharose beads (Sigma) for 1 hr. Separately, 30 μL of 50% G-sepharose beads (Sigma) were conjugate with 45M1 (0. 5 μL) antibody for 2 hrs. Immunoprecipitation was performed by incubation of the precleared lysate with the antibody conjugated beads for 3 hrs. Next, the beads were washed 5 times with washing buffer (1% w/v Triton X-100, 50 mM Tris-HCl pH 7.4, 300 mM NaCl, 5 mM EDTA, 0.02% w/v sodium azide) for maximal removal of unbound proteins. The immune complexes were released by boiling for 10 min in Laemmli buffer, the immunoprecipitates were loaded on a gel and Western blotting was performed as described above.

### Muc5ac isolation from mice stomach

Mucins were solubilized in 4 M guanidine chloride solution containing 5 mM EDTA, 10 mM benzamidine, 5 mM N-ethylmaleimide, 0.1 mg/mL soy bean trypsin inhibitor and 1 mM PMSF. CsCl was added to an initial density of 1.4 g/mL and mucins were purified by isopycnic density-gradient centrifugation (Beckman Coulter LE80K ultracentrifuge; 70.1 Ti rotor, 58000 rpm at 15 °C for 72 hrs). Fractions of 1 mL were collected from the bottom of the tube and analyzed for PAS reactivity, density, and reactivity against Muc5ac. The mucin-containing fractions were pooled, dialyzed into 4 M guanidinium chloride buffer and subjected to gel chromatography on Sepharose CL-4B. The column (85 × 2 cm *Pharmacia*) was eluted at a flow rate of 8 mL/hr with 4 M guanidinium chloride. Pooled fractions containing mucins were submitted to two centrifugation steps (30 min, 13000 g) in 4 M guanidine chloride. The pellet, containing Muc6, was solubilized in DTT (2 mM) during 1 hr at 37 °C in 6 M guanidine chloride and then alkylated with 5 mM iodoacetamide, 5 mM sodium EDTA, 10 mM Tris-HCl (pH 8) for 1 hr at 20 °C. Supernatant fractions, containing Muc5ac, were reduced and alkylated as described for the pellet. All fractions were dialyzed against water and lyophilized.

### Slot blot analysis of purified mucins

Aliquots of purified mucins were slot blotted onto polyvinylidene fluoride (PVDF) membranes. The membranes were then blocked with 0.5% (w/v) skimmed milk in TBS containing 0.1% (v/v) Tween 20 (blocking solution) for 1 hr and incubated with the polyclonal antibodies LUM6-3 and LUM5-1 recognizing MUC6 and MUC5AC, respectively^37^,^45^, at a dilution of 1:1000 in blocking solution. Bound antibody was detected by incubation with horseradish peroxidase-conjugated anti-rabbit (1:10000) antibody in blocking solution for 1 hr followed by the ECL Western detection kit. All incubations were carried out at room temperature.

### Dot blot analysis of purified Muc5ac

Approximately 80 mg of lyophilized Muc5ac purified from either wild-type or Fut2-null mice were resuspended in 100 μL of MQ-water. Serial dilutions were prepared (800; 80; 40; 20; 10 and 5 μg/μL) and 2 μL from each solution were sequentially applied as separate dots onto nitrocellulose membranes (Amersham Hybond-ECL Membrane, GE Healthcare, Buckinghamshire, UK). Membranes were blocked for 1 hr with PBS containing 5% BSA prior to incubation over-night at 4 °C with BG6, SPM279 and PM7 diluted in PBS with 1% BSA, as described in Table 1. As a negative control, primary antibody was replaced by PBS with 1% BSA. Blots were washed three times with PBS-Tween 0.01% and incubated with HRP-conjugated rabbit anti-mouse Igs antibody (DAKO, Glostrup, Denmark), diluted 1:1000 in PBS containing 1% BSA, for 1 hr at RT. Membranes were washed as described above and developed using ECL (Amersham ECL Western Blotting detection reagents, GE Healthcare, Buckinghamshire, UK).

### Release of oligosaccharides from mucin by alkaline borohydride treatment

The gastric mucins were submitted to β-elimination under reductive conditions. The mixture of oligosaccharide alditols was purified by size exclusion chromatography on a column of Bio*-*Gel P2 (85 × 2 cm ID, 400 mesh, Bio*-*Rad, Richmond, CA) equilibrated and eluted with water (10 mL/hr) at RT. The oligosaccharide fractions, detected by UV absorption at 206 nm, were pooled for structural analysis.

### Fractionation of the oligosaccharide alditols by HPLC

The mixture of oligosaccharide alditols released from each sample was subjected to fractionation by HPLC (Dionex Chromeleon System, Sunnyvale, CA, USA) on a primary amino-bonded silica column (Supelcosyl, LC-NH_2_, 4.6 × 250 mm, Supelco, Bellefonte, CA, USA). The column was equilibrated with the initial solvent using a mixture of acetonitrile/H_2_O (80:20, v/v) with a flow rate of 1 ml/min. After the injection, a linear gradient to 40:60, v/v for 80 min was applied followed by isocratic conditions for 20 min. Oligosaccharides were eluted with H_2_O. Oligosaccharides were detected by UV spectroscopy at 200 nm using an UVD 170 U detector (Dionex, Sunnyvale, CA, USA).

### Electrospray mass spectrometry (nanoESI-MS/MS)

All analyses were performed on a Q-STAR Pulsar quadrupole time-of-flight (Q-q-TOF) mass spectrometer (Applied Biosystems/MDS Sciex, Toronto, Canada) fitted with a nanoelectrospray ion source (Protana, Odense, Denmark). Oligosaccharides dissolved in water (60 pmol/μL) were acidified by addition of an equal volume of methanol/0.1% formic acid and sprayed from gold-coated “medium length” borosilicate capillaries (Protana). A potential of −800 V was applied to the capillary tip and the focusing potential was set at −100 V, the declustering potential varying between −60 V and −110 V. For the recording of conventional mass spectra, time-of-flight data were acquired by accumulation of 10 MCA (multiple channel acquisition) scans over mass ranges of m/z 400–2000. In the collision-induced dissociation (CID) tandem MS analyses, multiple charged ions were fragmented using nitrogen as collision gas (5.3 × 10^−5^ Torr), with the collision energy varying between −40 and −90 eV to obtain optimal fragmentation. The CID spectra were recorded on the orthogonal TOF analyzer over a range of m/z 80–2000. Data acquisition was optimized to supply the highest possible resolution and the best signal-to*-*noise ratio even in the case of low abundance signals. Typically, the full width at half maximum (FWHM) was 7000 in the measured mass ranges. External calibration was performed prior to each measure using a 4 pmol/μL solution of taurocholic acid in acetonitrile/water (50:50, v/v) containing 2 mM of ammonium acetate.

### Nuclear magnetic resonance (NMR) spectroscopy

Samples were repeatedly treated with ^2^H_2_O (99.97% ^2^H atoms, Euriso*-top*, CEA, Saclay, France) and transferred in 200 × 5-mm BMS-005B Shigemi® tubes matched for D_2_O. Chemical shifts were expressed in parts/million (ppm) and calibrated using internal acetone D6 (δ^1^H 2.225 and δ^13^C 31.55 ppm). Spectra were acquired at 300 K on the 14.3Teslas spectrometer equipped with CPQCI (^1^H, ^15^N, ^13^C, ^19^F) cryo-probe head (Bruker®, located in Pasteur Institute) where ^1^H resonated at 600.13 MHz and ^13^C at 150.9 MHz. To better determine characteristic reporter groups, different spectra were recorded such as ^1^H-1D, ^1^H-^1^H COSY, ^1^H-^1^H-TOCSY and ^1^H-^13^C HSQC NMR spectra.

### Bacterial strains

*H. pylori* reference strain 17875/Leb^23^ were grown on Brucella agar medium (BD, Stockholm, Sweden) with 10% bovine blood and 1% IsoVitox (Svenska labfab, Ljusne, Sweden) at 37 °C under microaerobic conditions. *Escherichia coli* (*E. coli*) XL 10 Gold (Stratagene, USA) was used as negative control.

### Muc5ac-*H. pylori* binding assessed by Radio immuno assay (RIA)

Purified Muc5ac from wild-type and Fut2-null mice was radiolabeled with ^125^I (Perkin-Elmer NEZ033A005MC) using the Chloramine method^46^ and RIA analysis was performed as previously described^47^ with the following modifications. Prior to the addition of bacteria to the radiolabeled Muc5ac, a pre-incubation of Muc5ac with blocking buffer (1% BSA in PBS containing 0.05% Tween 20) was performed, for 1 hr at RT, to block potential unspecific binding sites of the Muc5ac molecule. Then Muc5ac was mixed with bacteria (0.1 optical density at A600 nm) over-night with gentle agitation at RT. Bacterial cells pellets were obtained by 15 minutes centrifugation at 20,000 g, and the supernatant and pellet were counted separately in a gamma scintillation counter.

### Histobinding assay of native *H. pylori* BabA adhesin

BabA protein was purified from 17875Leb *H. pylori* cells by affinity chromatography (Bugaytsova *et al.*, in preparation). Mice gastric mucosal tissue sections were deparaffinized, rehydrated and incubated for 3 hrs with 2% BSA in PBS-T (PBS with 0.05% Tween 20). After this blocking step, tissues were briefly washed with PBS to remove excess of BSA and Tween 20. Tissue sections were then incubated with purified native BabA protein at a concentration of 2 μg/mL in PBS over-night at 4 °C. A negative control was included in all experiments that consists in pre-incubation of BabA with 10 μg/mL Leb-HSA conjugate (IsoSep AB, Tullinge, Sweden) for 1 hr in PBS-T at RT, prior to incubation with the tissue sections. Tissue sections were washed three times with PBS-T and slides were incubated for 3 hrs at RT with BabA specific antibody Vite diluted 1:4000 in PBS-T (Table 1). Then, sections were washed as described before and incubated with biotinylated mouse anti-rabbit antibody (DAKO, Glostrup, Denmark) diluted 1:2000 in PBS-T for 1 hr at RT. Following three wash steps with PBS-T, tissues were incubated in the dark with streptavidin Alexa Fluor 555 conjugate (Invitrogen, Life Technologies) diluted 1:100 in PBS-T for 30 min. Finally, sections were then washed with PBS and incubated for 5 minutes with DAPI, in the dark. Samples were washed in PBS and mounted using DAKO fluorescent mounting media (DAKO, Glostrup, Denmark). Images were acquired using a Zeiss Axio cam MRm and the Zen Blue software.

## Additional Information

**How to cite this article**: Magalhães, A. *et al.* Muc5ac gastric mucin glycosylation is shaped by FUT2 activity and functionally impacts *Helicobacter pylori* binding. *Sci. Rep.* **6**, 25575; doi: 10.1038/srep25575 (2016).

## Supplementary Material

Supplementary Information

## Figures and Tables

**Table 1:** Specificity and dilutions of antibodies used for immunofluorescence and Western blot analysis.

Antibody clone	Ig isotype	Working dilution		Antigen (Reference)
		IF	WB	
45M1	IgG1	1:1000	1:1000	Muc5ac^36^
PM7^a^	–	–	1:5	Muc5ac
7LE	IgG1	1:5	–	Lewis a^48^,^49^
SPM279 (Santa Cruz)	IgG1	–	1:200	Lewis a
BG6 (Signet T218)	IgM	1:50	1:200	Lewis b^50^
Vite			–	BabA^b^

^a^Mixture of equal volume of 1–13M1, 2–11M1, 2–12M1, 9–13M1, 19M1, 21M1, 45M1.

^b^Antibody Vite was produced at the T. Borén laboratorium according to the previously described protocol^51^.
