# Supplementary material for: Muc5ac gastric mucin glycosylation is shaped by FUT2 activity and functionally impacts Helicobacter pylori binding
Source: Sci Rep. 2016 May 10;6:25575. doi: 10.1038/srep25575 (PMC4861914; doi:10.1038/srep25575)
Supplement: Supplementary Information [file srep25575-s1.pdf]

Muc5ac gastric mucin glycosylation is shaped by FUT2 activity and functionally impacts *Helicobacter pylori* binding

Ana Magalhães, Yannick Rossez, Catherine Robbe-Masselot, Emmanuel Maes, Joana Gomes, Anna Shevtsova, Jeanna Bugaytsova, Thomas Borén, and Celso A. Reis

Supplementary Information

Supplementary Methods

1. Quantitative real-time PCR analysis of *Muc5ac* and *Muc6*

Total RNA was extracted from 7-9 week old wild-type and Fut2-null mice stomachs using TRI reagent LS (Sigma-Aldrich), according to manufacturer's protocol. RNA yield and quality were determined spectrophotometrically and 5.0µg of total RNA was reverse transcribed using Superscript III (Invitrogen), according to manufacturer's instructions. Expression of *Muc5ac* and *Muc6* was quantified using Taqman probes, acquired as pre-developed assays from Applied Biosystems (Mm01276718_m1 and Mm00725165_m1) and normalized to the expression of the endogenous control *Hprt* (Mm03024075_m1). Each sample was amplified in triplicate in an ABI Prism 7500 (Applied Biosystems). Relative transcript levels were determined using the ΔΔCT-method.

Supplementary Figure S1

***Muc5ac* and *Muc6* transcript levels in wild-type and Fut2-null mice gastric mucosa**

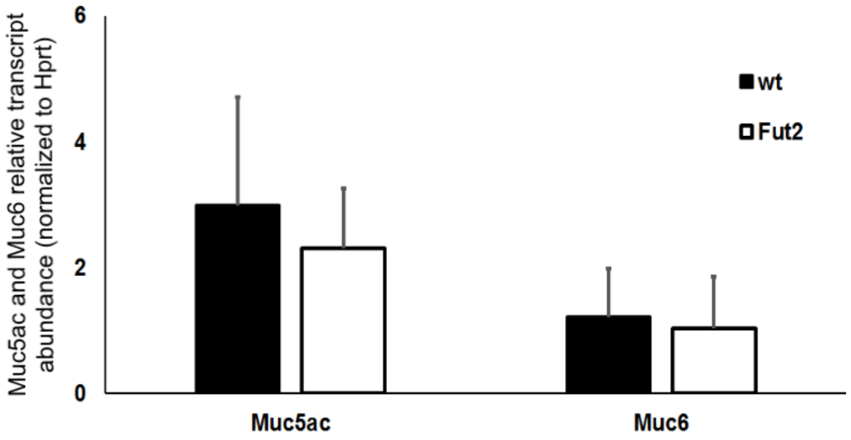

Supplementary Figure S1 - Gastric mucosal transcript levels of *Muc5ac* and *Muc6* in wild type and Fut2-null mice. qRT-PCR analysis of *Muc5ac* and *Muc6* genes. qRT-PCR reactions were performed in triplicate for three independent mice samples and relative transcript abundance of the target gene was normalized to *Hprt* expression levels.
